# Prevalence of and Risk Factors for Adhesive Capsulitis of the Shoulder in Older Adults from Germany

**DOI:** 10.3390/jcm12020669

**Published:** 2023-01-14

**Authors:** Louis Jacob, Razak M. Gyasi, Ai Koyanagi, Josep Maria Haro, Lee Smith, Karel Kostev

**Affiliations:** 1 Research and Development Unit, Parc Sanitari Sant Joan de Déu, CIBERSAM, ISCIII, Dr. Antoni Pujadas, 42, Sant Boi de Llobregat, 08830 Barcelona, Spain; 2 Department of Physical Medicine and Rehabilitation, Lariboisière-Fernand Widal Hospital, AP-HP, University Paris Cité, 75010 Paris, France; 3 African Population and Health Research Center, Nairobi 00100, Kenya; 4 National Centre for Naturopathic Medicine, Faculty of Health, Southern Cross University, Lismore, NSW 2480, Australia; 5 Institució Catalana de Recerca i Estudis Avançats (ICREA), Pg. Lluis Companys 23, 08010 Barcelona, Spain; 6 Centre for Health, Performance and Wellbeing, Anglia Ruskin University, Cambridge CB1 1PT, UK; 7 Epidemiology, IQVIA, 60549 Frankfurt, Germany

**Keywords:** adhesive capsulitis, frozen shoulder, epidemiology, older adults, Germany

## Abstract

This study aimed to investigate the prevalence of and risk factors for adhesive capsulitis in older adults from Germany. The one-year and lifetime prevalence of adhesive capsulitis were assessed in adults aged ≥65 years with at least one visit to one of 1207 general practices in Germany in 2021. Associations between physical and psychiatric conditions and adhesive capsulitis were further assessed in older patients diagnosed for the first time with adhesive capsulitis in general practices in 2010–2021 (index date) and matched (1:5) to patients without adhesive capsulitis using a propensity score based on age, sex, and the index year. In adults without adhesive capsulitis, the index date was a randomly selected visit date in 2010–2021. The one-year and lifetime prevalence of adhesive capsulitis in older adults in 2021 were 0.4% and 2.4%, respectively. In 8439 patients with and 42,195 patients without adhesive capsulitis, 12 conditions were positively and significantly associated with adhesive capsulitis. Effect sizes were strongest for other and unspecified osteoarthritis (OR = 1.93), injury to the shoulder and upper arm (OR = 1.85), and injury to the thorax (OR = 1.47). Based on these findings, adhesive capsulitis can occur at older age, and osteoarthritis and injuries are major risk factors.

## 1. Introduction

Adhesive capsulitis of the shoulder (i.e., frozen shoulder) is a chronic shoulder condition characterized by pain, loss of range of motion, and decreased upper arm function [1]. Usually, adhesive capsulitis progresses through three distinct phases (i.e., painful, stiffness, and recovery phases) and lasts between one and three years [2]. The diagnosis of adhesive capsulitis relies on physical examination, and imaging studies are only necessary to exclude differential diagnoses (e.g., glenohumeral osteoarthritis, rotator cuff tendinopathy, and autoimmune disease) [3]. Adhesive capsulitis has a negative impact on mental health [4], quality of life [5], and work productivity [6]. In this context, it is of the utmost importance to better understand the epidemiology of adhesive capsulitis.

The prevalence of adhesive capsulitis of the shoulder is between 3% and 5% in the general population [2]. The majority of patients are aged between 40 and 59 years at diagnosis [7], but some evidence suggests that adhesive capsulitis can also occur later in life. A study of more than 26 million people aged ≥65 years from the United States revealed that the one-year prevalence of adhesive capsulitis was 0.35% [8]. Several conditions have been identified as risk factors for adhesive capsulitis, including, for example, diabetes, disorders of the thyroid gland, and cerebrovascular diseases [2]. Although the previous studies focusing on risk factors for adhesive capsulitis have advanced the field, samples were mainly constituted of middle-aged adults [9,10,11,12]. Findings obtained in middle-aged individuals may not be extrapolated to the older adult population, as the comorbidity profile of patients may change with age [13,14]. Moreover, effect sizes for the association between some chronic conditions and adhesive capsulitis may be different between middle and older age groups. For example, given that there is a positive relationship between glycated hemoglobin (HbA1c) and adhesive capsulitis in individuals with diabetes [15], and that HbA1c increases with age [16], the association between diabetes and adhesive capsulitis may be stronger in older adults compared to those of middle age. Taken together, little is known about the epidemiology of adhesive capsulitis in older adults, highlighting the need for further data on this topic.

Therefore, the present retrospective study aimed to investigate the prevalence of and risk factors for adhesive capsulitis of the shoulder in patients aged ≥65 years from general practices in Germany.

## 2. Materials and Methods

### 2.1. Database

This study used data from the Disease Analyzer database (IQVIA). Details on the methodology of the database have been published elsewhere [17]. Briefly, the Disease Analyzer database contains data on demographic variables, diagnoses, and prescriptions obtained in general and specialized practices in Germany. The International Classification of Diseases, 10th revision (ICD-10), is used to code diagnoses, while the Anatomical Classification of Pharmaceutical Products of the European Pharmaceutical Market Research Association (EphMRA) is used to code prescriptions. Data quality is assessed monthly based on several criteria, such as completeness of documentation and linkage between diagnoses and prescriptions. The selection of general and specialized practices to include in the Disease Analyzer database relies on the statistics of the German Medical Association, which are published every year and provide data on the physician’s age, specialty group, community size category, and German federal state. Finally, the Disease Analyzer database includes approximately 3% of all practices in Germany and is representative of these practices with relatively similar characteristics (e.g., distribution of the age and sex of patients and prevalence of chronic physical conditions) [17].

### 2.2. Study Populations

**Figure 1:**
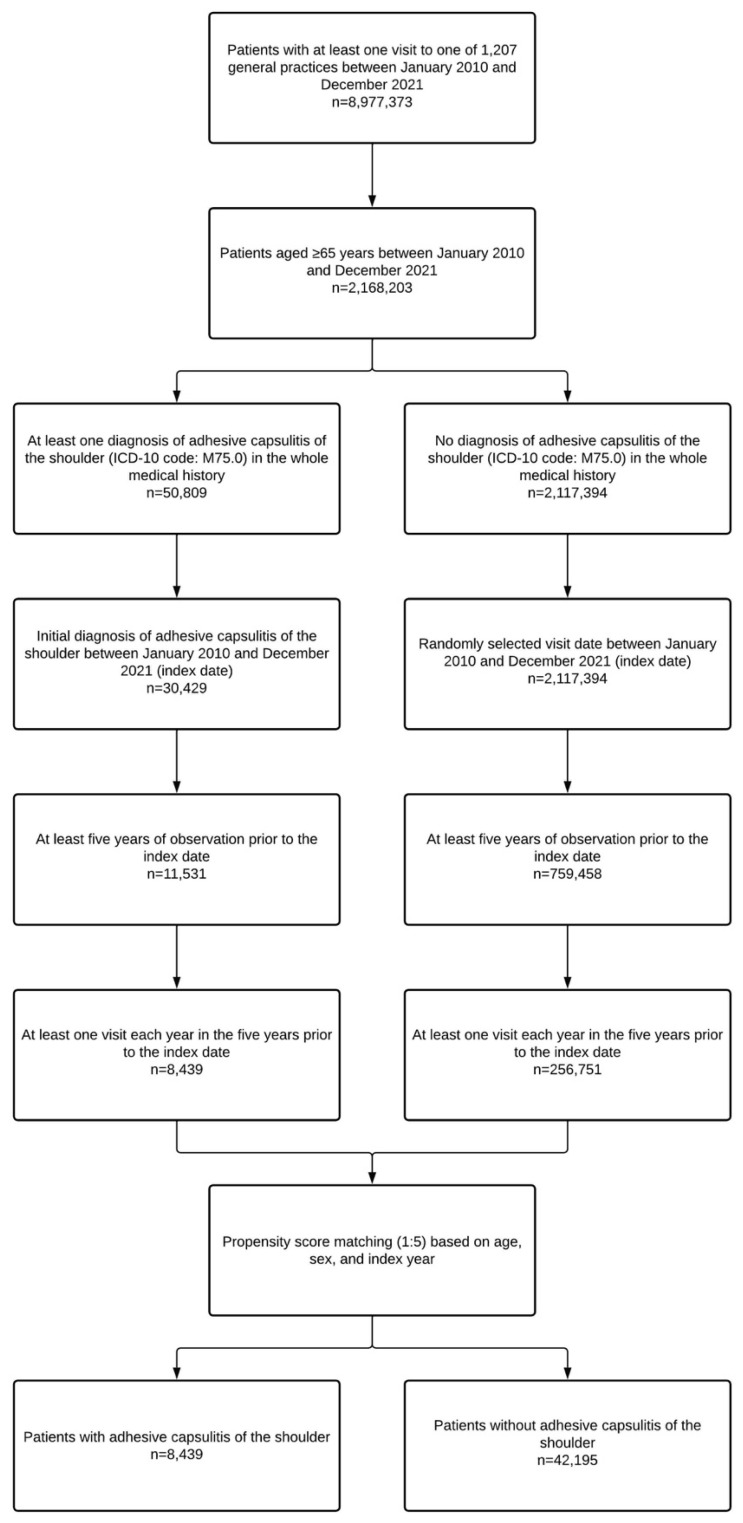
Flow diagram of study patients. Abbreviation: ICD-10, International Classification of Diseases, 10th revision.

Two distinct populations were included in the present retrospective study. The first population was used to estimate the one-year and lifetime prevalence of adhesive capsulitis of the shoulder in older adults from general practices in Germany in 2021. This population included all individuals aged ≥65 years with at least one visit to one of 1207 general practices in 2021. The second population was used to identify physical and psychiatric conditions significantly associated with adhesive capsulitis. This population included all patients aged ≥65 years with an initial diagnosis of adhesive capsulitis of the shoulder (ICD-10 code: M75.0) between January 2010 and December 2021 (index date; age was assessed at the index date). Additional inclusion criteria were at least five years of observation prior to the index date and at least one visit each year in the five years prior to the index date. After applying similar inclusion criteria, patients aged ≥65 years without adhesive capsulitis were matched (1:5) to those with adhesive capsulitis using a propensity score based on age, sex, and index year. In participants without adhesive capsulitis of the shoulder, the index date was a randomly selected visit date between January 2010 and December 2021. The flow diagram of the second study population is displayed in Figure 1.

### 2.3. Physical and Psychiatric Conditions

Physical and psychiatric conditions were documented in the five years prior to the index date. These conditions were selected based on previous literature and included cancer (ICD-10 codes: C00-C97) [18], disorders of the thyroid gland (ICD-10 codes: E00-E07) [19], diabetes (ICD-10 codes: E10-E14) [20], obesity (ICD-10 code: E66) [21], disorders of lipoprotein metabolism and other lipidemias (ICD-10 code: E78) [22], depression (ICD-10 codes: F32 and F33) [23], anxiety disorders (ICD-10 code: F41) [23], reaction to severe stress and adjustment disorders (ICD-10 code: F43) [23], somatoform disorders (ICD-10 code: F45) [23], Parkinson’s disease (ICD-10 code: G20) [8], hypertension (ICD-10 code: I10) [24], ischemic heart diseases (ICD-10 codes: I20-I25) [2], cerebrovascular diseases (ICD-10 codes: I60-I69) [2], unspecified chronic bronchitis (ICD-10 code: J42) [25], gastro-esophageal reflux disease (ICD-10 code: K21) [26], rheumatoid arthritis (ICD-10 codes: M05 and M06) [24], gout (ICD-10 code: M10) [27], polyosteoarthritis (ICD-10 code: M15) [24], other and unspecified osteoarthritis (ICD-10 code: M19) [24], palmar fascial fibromatosis (Dupuytren’s disease; ICD-10 code: M72.0) [12], injuries to the thorax (ICD-10 codes: S20-S29) [24], and injuries to the shoulder and upper arm (ICD-10 codes: S40-S49) [24].

### 2.4. Statistical Analyses

The one-year prevalence of adhesive capsulitis of the shoulder was estimated as the proportion of individuals diagnosed at least once with adhesive capsulitis in 2021 among patients with at least one visit to their general practice in 2021. Similarly, the lifetime prevalence of adhesive capsulitis corresponded to the proportion of individuals diagnosed at least once with adhesive capsulitis in their whole medical history among patients with at least one visit to their general practice in 2021. Demographic characteristics of patients without adhesive capsulitis in 2010–2021 matched to those with adhesive capsulitis in 2010–2021 were compared using the Wilcoxon signed-rank test for continuous age, the Stuart–Maxwell test for categorical age, and the McNemar test for sex. Finally, the associations between physical and psychiatric conditions (independent variables) and the diagnosis of adhesive capsulitis of the shoulder in 2010–2021 (dependent variable) were studied using an adjusted logistic regression model. All physical and psychiatric diseases were simultaneously included in the logistic regression model. Results of the logistic regression model are displayed as odds ratios (ORs) and 95% confidence intervals (CIs). *p*-values < 0.001 were considered statistically significant. All analyses were performed using SAS 9.4.

## 3. Results

### 3.1. One-Year and Lifetime Prevalence of Adhesive Capsulitis of the Shoulder

**Figure 2:**
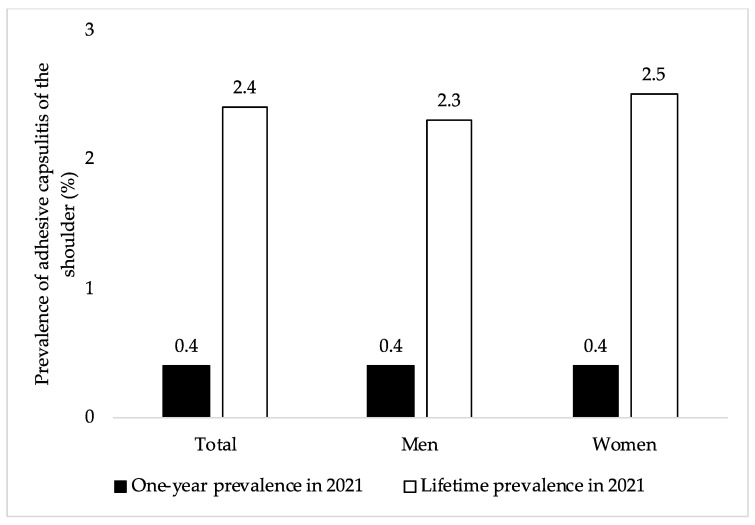
One-year and lifetime prevalence of adhesive capsulitis of the shoulder in 2021 in this sample of adults aged ≥65 years from Germany (overall and by sex). Abbreviation: ICD-10, International Classification of Diseases, 10th revision. One-year prevalence of adhesive capsulitis of the shoulder was defined as the proportion of individuals diagnosed at least once with adhesive capsulitis (ICD-10 code: M75.0) in 2021 among patients with at least one visit to their general practice in 2021. Lifetime prevalence of adhesive capsulitis of the shoulder was defined as the proportion of individuals diagnosed at least once with adhesive capsulitis in their whole medical history among patients with at least one visit to their general practice in 2021.

The one-year and lifetime prevalence of adhesive capsulitis of the shoulder in the overall sample and by sex are displayed in Figure 2. Among 859,166 patients aged ≥65 years with at least one visit to one of 1207 general practices in Germany in 2021, the one-year and lifetime prevalence of adhesive capsulitis were 0.4% and 2.4%, respectively. Similar findings were obtained in men and women.

### 3.2. Demographic Characteristics at the Index Date of Patients with and without Adhesive Capsulitis of the Shoulder

After 1:5 matching of patients with adhesive capsulitis of the shoulder with those without adhesive capsulitis using a propensity score based on age, sex, and index year, there were 8439 participants in the adhesive capsulitis group and 42,195 participants in the no adhesive capsulitis group. Mean (standard deviation) age at the index date was 75.3 (6.8) years, while the prevalence of women was 57.4% (Table 1).

### 3.3. Physical and Psychiatric Conditions Associated with Adhesive Capsulitis of the Shoulder

The results of the adjusted logistic regression model are shown in Table 2. There were 12 physical and psychiatric conditions positively and significantly associated with the diagnosis of adhesive capsulitis of the shoulder between 2010 and 2021. These conditions were documented in the five years prior to the index date (i.e., the first diagnosis of adhesive capsulitis) and included other and unspecified osteoarthritis (OR = 1.93, 95% CI = 1.79–2.07), injury to the shoulder and upper arm (OR = 1.85, 95% CI = 1.66–2.05), injury to the thorax (OR = 1.47, 95% CI = 1.34–1.61), polyosteoarthritis (OR = 1.44, 95% CI = 1.32–1.57), palmar fascial fibromatosis (Dupuytren’s disease; OR = 1.41, 95% CI = 1.17–1.70), gout (OR = 1.36, 95% CI = 1.26–1.47), reaction to severe stress and adjustment disorders (OR = 1.32, 95% CI = 1.21–1.45), gastro-esophageal reflux disease (OR = 1.29, 95% CI = 1.22–1.36), somatoform disorders (OR = 1.19, 95% CI = 1.11–1.28), obesity (OR = 1.15, 95% CI = 1.06–1.24), ischemic heart diseases (OR = 1.13, 95% CI = 1.07–1.19), and disorders of the thyroid gland (OR = 1.11, 95% CI = 1.06–1.17). In contrast, there was a negative and significant relationship between Parkinson’s disease (OR = 0.71, 95% CI = 0.60–0.85), hypertension (OR = 0.81, 95% CI = 0.77–0.86), and adhesive capsulitis.

## 4. Discussion

### 4.1. Main Findings

The one-year and lifetime prevalence of adhesive capsulitis of the shoulder in adults aged ≥65 years from general practices in Germany in 2021 were 0.4% and 2.4%, respectively. The adjusted logistic regression model conducted on 8439 adults with and 42,195 adults without adhesive capsulitis further revealed that 12 physical and psychiatric conditions were positively and significantly associated with the diagnosis of adhesive capsulitis in 2010–2021. These conditions were other and unspecified osteoarthritis (OR = 1.93), injury to the shoulder and upper arm (OR = 1.85), injury to the thorax (OR = 1.47), polyosteoarthritis (OR = 1.44), palmar fascial fibromatosis (Dupuytren’s disease; OR = 1.41), gout (OR = 1.36), reaction to severe stress and adjustment disorders (OR = 1.32), gastro-esophageal reflux disease (OR = 1.29), somatoform disorders (OR = 1.19), obesity (OR = 1.15), ischemic heart diseases (OR = 1.13), and disorders of the thyroid gland (OR = 1.11). Finally, there was a negative and significant relationship between adhesive capsulitis and Parkinson’s disease (OR = 0.71) and hypertension (OR = 0.81). To the best of the authors’ knowledge, this is only the second study to investigate the epidemiology of adhesive capsulitis in the older population, while this research is the first to analyze the effects of a large number of physical and psychiatric disorders on adhesive capsulitis, particularly in older age.

### 4.2. Interpretation of Findings

In this retrospective study of older patients followed in general practices in Germany, the one-year prevalence of adhesive capsulitis of the shoulder was 0.4%. The finding was corroborated in the sex-stratified analyses. This finding is in line with the sole study on the epidemiology of adhesive capsulitis in older adults, which reported a one-year prevalence of 0.35% in the United States [8]. Given that approximately 18.4 million people were aged ≥65 years in Germany in 2021 [28], the present proportion translates into more than 73,000 new diagnoses of adhesive capsulitis each year in the older population living in Germany. These data clearly show that adhesive capsulitis affects not only working-age adults but also their older counterparts, highlighting the need for tailored treatment and management of this shoulder disorder in later life.

There was a positive and significant association between several physical and psychiatric conditions and adhesive capsulitis of the shoulder. Osteoarthritis (i.e., other and unspecified osteoarthritis and polyosteoarthritis) was strongly associated with adhesive capsulitis. Although it was not possible in this study to differentiate shoulder osteoarthritis from osteoarthritis of other regions of the body (e.g., elbow osteoarthritis), it is likely that the observed associations are largely explained by shoulder osteoarthritis. Interestingly, in a population-based nested case–control study of 24,414 individuals with and 97,656 individuals without adhesive capsulitis from Taiwan, osteoarthritis was associated with a 4.27-fold increase in the odds of adhesive capsulitis after adjusting for multiple confounding variables [24]. It was suggested that osteoarthritis-related shoulder function impairments might partially explain the osteoarthritis–adhesive capsulitis relationship. There was also a substantial association between injury to the shoulder and upper arm, injury to the thorax, and adhesive capsulitis, corroborating the findings of previous literature [24]. As for osteoarthritis, injuries may lead to shoulder dysfunction, which may, in turn, favor the occurrence of adhesive capsulitis.

Palmar fascial fibromatosis, also known as Dupuytren’s disease, was positively and significantly associated with adhesive capsulitis. Palmar fascial fibromatosis, which corresponds to the benign proliferation of fibroblasts and myofibroblasts, involves nodules and contractures on the palmar crease [29]. A case–control study, including 263 patients presenting to a single center in Australia, found that palmar fascial fibromatosis tended to be more frequent in the adhesive capsulitis than in the no adhesive capsulitis group (8% versus 3%, *p*-value = 0.068), the lack of statistical significance potentially being related to the small sample size [12]. In addition, a recent genome-wide association study based on data from the UK Biobank identified multiple loci significantly associated with both palmar fascial fibromatosis and adhesive capsulitis, suggesting that the two disorders may have a shared genetic architecture [30].

The present retrospective study from Germany further showed a significant association between gout and adhesive capsulitis of the shoulder. Findings pointing in the same direction were obtained in a Taiwanese nationwide population-based matched-cohort study of 117,282 adults aged 40–70 years, as gout led to a 1.71-fold increase in the adjusted risk of adhesive capsulitis [27]. Gout is a common inflammatory arthritis caused by the deposition of monosodium urate crystals within joints [31]. The relationship between gout and adhesive capsulitis likely involves several mediators, such as low-grade inflammation of the shoulder, hyperuricemia, and several chronic conditions (e.g., cardiovascular diseases). Another physical condition significantly associated with adhesive capsulitis was gastro-esophageal reflux disease. It was observed in an Italian sample of 237 patients undergoing arthroscopic rotator cuff repair that the presence of gastro-esophageal reflux disease positively predicted the occurrence of postoperative shoulder stiffness [26]. The effects of gastro-esophageal reflux disease on adhesive capsulitis may be partially explained by inflammation and the malabsorption of retinoids, with retinoid metabolism being potentially disrupted in the capsules of individuals with adhesive capsulitis [32].

This research also showed that participants with obesity were more likely to be diagnosed with adhesive capsulitis of the shoulder than their counterparts without obesity. This result is in line with another study of 4380 patients from the United States, as obesity was significantly more frequent in people with than in those without adhesive capsulitis [21]. Although the mechanisms underlying the obesity–adhesive capsulitis relationship are insufficiently understood, one hypothesis is that low-grade inflammation plays a decisive role in the association. Finally, ischemic heart diseases and disorders of the thyroid gland were identified as predictors of adhesive capsulitis, corroborating a solid body of literature on the topic [2]. Ischemic heart diseases may increase the risk of adhesive capsulitis via impaired circulation [7], while proinflammatory cytokines and fibroblast proliferation may mediate the association between thyroid gland disorders and adhesive capsulitis [33].

In terms of psychiatric conditions, two disorders (i.e., reaction to severe stress and adjustment disorders, and somatoform disorders) were positively and significantly associated with adhesive capsulitis of the shoulder. This finding is relatively new and should be interpreted with caution. Interestingly, previous research has reported a high prevalence of psychiatric conditions in patients with adhesive capsulitis compared with the general population [4]. The present result adds to the literature by showing that poor mental health may predispose individuals to adhesive capsulitis. Furthermore, although the level of evidence is low, multiple psychological factors (e.g., emotional distress, somatization, and fear-avoidance beliefs) may play a key role in the perpetuation of pain intensity and disability among individuals with chronic shoulder pain [23].

Finally, there was a negative and significant association between Parkinson’s disease, hypertension, and adhesive capsulitis, while no statistically significant relationships were observed for diabetes and cerebrovascular diseases. The negative Parkinson’s disease–adhesive capsulitis relationship is unexpected, as a previous review article has obtained opposite findings [34]. Given that patients with Parkinson’s disease may have also been followed in neurological practices, one may hypothesize that adhesive capsulitis was documented in neurological and not in general practices, potentially biasing the present association. The relationship between hypertension and adhesive capsulitis remains under debate, as previous studies have reported positive [24] and null associations [8,11,35]. In this context, more data are warranted on the potential hypertension–adhesive capsulitis relationship. The lack of significant association between diabetes, cerebrovascular diseases, and adhesive capsulitis should also be pointed out. In the case of diabetes, the effect size was almost significant, and significant results might have been obtained with a larger sample size. In addition, patients with diabetes included in this study might display a mild form of the disease, and those with moderate and severe forms, who are at particular risk for adhesive capsulitis [15], might have been followed in specialized practices (e.g., diabetology practices). In the case of cerebrovascular diseases, this is one of the first studies to include a large number of physical and psychiatric conditions, and the cerebrovascular disease–adhesive capsulitis relationship reported in the literature [2] may largely be explained by comorbidities of cerebrovascular diseases.

### 4.3. Clinical Implications and Directions for Future Research

Based on these findings, adhesive capsulitis of the shoulder affects not only middle-aged but also older adults. Conditions the most strongly associated with adhesive capsulitis were osteoarthritis and injuries to the shoulder, upper arm, and thorax. When these conditions are present, shoulder pain, range of motion, and function should be regularly assessed by general practitioners. The first-line treatment of adhesive capsulitis is physiotherapy, and several physiotherapy interventions (e.g., stretching exercises, mobilization, and strengthening of muscles) are recommended for the management of this shoulder condition [36]. Corticosteroid injections should be prescribed with caution in older patients, as several disorders (e.g., uncontrolled diabetes, uncontrolled hypertension, and urinary infection) may counter-indicate these injections. In terms of future research, more studies are warranted to corroborate or invalidate the present results in other countries and settings.

### 4.4. Strengths and Limitations

Strengths of the study are the large sample size, the use of data collected in general practices, and the inclusion of a wide range of physical and psychiatric conditions. Nonetheless, the study findings should be interpreted in light of several limitations. First, the diagnosis of adhesive capsulitis exclusively relied on the ICD-10 classification. More data on adhesive capsulitis (e.g., severity and duration of symptoms) would have allowed more detailed analyses. Second, adhesive capsulitis may have been diagnosed in rheumatology practices, and the prevalence of this shoulder condition may have been underestimated. Third, there was no information on prolonged immobilization of the shoulder and HLA-B27 status, and given that these factors may be associated with adhesive capsulitis of the shoulder [25,37], residual confounding may exist. Fourth, patients living in institutions were not included in the study, and the findings cannot, therefore, be generalized to this setting.

## 5. Conclusions

This retrospective study, including older adults followed in general practices in Germany, found that the one-year and lifetime prevalence of adhesive capsulitis of the shoulder were 0.4% and 2.4%, respectively. Multiple physical and psychiatric conditions were significantly associated with adhesive capsulitis, and effect sizes were the strongest for osteoarthritis and injuries to the shoulder, upper arm, and thorax. More studies on the epidemiology of adhesive capsulitis in older adults are needed to confirm the present results.

## Figures and Tables

**Table 1:** Demographic characteristics of the study patients after 1:5 matching.

Variable	Adhesive Capsulitis of the Shoulder (n = 8439)	No Adhesive Capsulitis of the Shoulder (n = 42,195)	*p*-Value
Age (in Years) at the Index Date			
Mean (standard deviation)	75.3 (6.8)	75.3 (6.8)	0.856
65–70	2451 (29.0)	12,430 (29.5)	0.935
71–75	2032 (24.1)	9939 (23.6)	
76–80	1955 (23.2)	9739 (23.1)	
>80	2001 (23.7)	10,087 (23.9)	
Sex			
Female	4841 (57.4)	24,204 (57.4)	1.000
Male	3598 (42.6)	17,991 (42.6)	

Data are N (%) unless otherwise stated. Patients without adhesive capsulitis of the shoulder were matched to those with adhesive capsulitis of the shoulder using a propensity score based on age, sex, and index year. *p*-values were obtained using the Wilcoxon signed-rank test for continuous age, the Stuart–Maxwell test for categorical age, and the McNemar test for sex.

**Table 2:** Physical and psychiatric conditions significantly associated with adhesive capsulitis of the shoulder in patients aged ≥65 years from Germany (adjusted logistic regression model).

Condition	Patients with Adhesive Capsulitis of the Shoulder	Patients without Adhesive Capsulitis of the Shoulder	Odds Ratio	95% Confidence Interval	*p*-Value
Other and unspecified osteoarthritis	1281 (15.2)	3163 (7.5)	1.93	1.79–2.07	<0.001
Injury to the shoulder and upper arm	554 (6.6)	1354 (3.2)	1.85	1.66–2.05	<0.001
Injury to the thorax	702 (8.3)	2176 (5.2)	1.47	1.34–1.61	<0.001
Polyosteoarthritis	800 (9.5)	2429 (5.8)	1.44	1.32–1.57	<0.001
Palmar fascial fibromatosis (Dupuytren’s disease)	154 (1.8)	496 (1.2)	1.41	1.17–1.70	<0.001
Gout	903 (10.7)	3214 (7.6)	1.36	1.26–1.47	<0.001
Reaction to severe stress and adjustment disorders	763 (9.0)	2582 (6.1)	1.32	1.21–1.45	<0.001
Gastro-esophageal reflux disease	2200 (26.1)	8416 (19.9)	1.29	1.22–1.36	<0.001
Somatoform disorders	1348 (16.0)	5030 (11.9)	1.19	1.11–1.28	<0.001
Obesity	982 (11.6)	3922 (9.3)	1.15	1.06–1.24	<0.001
Rheumatoid arthritis	443 (5.2)	1604 (3.8)	1.15	1.03–1.28	0.015
Unspecified chronic bronchitis	314 (3.7)	1187 (2.8)	1.14	1.00–1.30	0.054
Ischemic heart diseases	2866 (34.0)	12,692 (30.1)	1.13	1.07–1.19	<0.001
Disorders of thyroid gland	2859 (33.9)	12,741 (30.2)	1.11	1.06–1.17	<0.001
Diabetes	3081 (36.5)	14,316 (33.9)	1.06	1.00–1.11	0.042
Depression	1896 (22.5)	8266 (19.6)	1.01	0.95–1.07	0.837
Cancer	1731 (20.5)	8167 (19.4)	1.00	0.94–1.06	0.960
Anxiety disorders	661 (7.8)	2717 (6.4)	1.00	0.91–1.10	0.975
Disorders of lipoprotein metabolism and other lipidemias	3729 (44.2)	18,144 (43.0)	0.97	0.92–1.02	0.212
Cerebrovascular diseases	1386 (16.4)	6606 (15.7)	0.97	0.91–1.04	0.414
Hypertension	5926 (70.2)	30,487 (72.3)	0.81	0.77–0.86	<0.001
Parkinson’s disease	165 (2.0)	1032 (2.4)	0.71	0.60–0.85	<0.001

Data are N (%) unless otherwise stated. The associations between physical and psychiatric conditions and adhesive capsulitis of the shoulder were studied using an adjusted logistic regression model. All physical and psychiatric diseases were simultaneously included in the logistic regression model. *p*-values < 0.001 were considered statistically significant.

## Data Availability

The data that support the findings of this study are available from the corresponding author upon reasonable request.
